# Nano-modified biosensors for detection of pathogenic diseases: The prospect of smart, multiplex and point-of-care testing

**DOI:** 10.5599/admet.2799

**Published:** 2025-07-09

**Authors:** Abdullahi Umar Ibrahim, Pwadubashiyi Coston Pwavodi, Mehmet Oszoz, Basil Barth Duwa, Irkham Irkham, Yeni Wahyuni Hartati

**Affiliations:** 1 Department of Biomedical Engineering, Near East University, Nicosia, Mersin 10, Turkey; 2 Department of Medical Biochemistry, Kaduna State University, Kaduna, Nigeria; 3 Department of Bioengineering/Biomedical/Medical Engineering, Faculty of Engineering, Cyprus International University, Haspolat, 99258, Nicosia, North Cyprus, Turkey; 4 Department of Chemistry, Faculty of Mathematics and Natural Sciences, Padjadjaran University, Indonesia; 5 Study Centre of Sensors and Green Chemistry, Faculty of Mathematics and Natural Sciences, Padjadjaran University, Indonesia

**Keywords:** Nanobiosensors, infectious diseases, smart technology, immunosensors, genosensors, electrochemical, optical

## Abstract

**Introduction and background:**

The world has witnessed several outbreaks, emergence and re-emergence of infectious diseases throughout the 21^st^ century as a result of climate change, urbanization and migration. Several infectious diseases caused by pathogens such as SARS-CoV-2, Ebola, Zika, Dengue, Marburg viruses, *Mycobacterium tuberculosis*, etc. have caused a devastating impact on lives and livelihoods around the world. To counter these diseases, medical experts rely on conventional techniques, which include microscopy and serological testing. However, these conventional methods are hindered by several trade-offs, including high cost, longer processing times, low sensitivity, and a likelihood of false positive results. Biomedical sensors have gained momentum in clinical diagnostics due to their low cost, portability, and sensitivity, among other advantages. To improve their performance, scientists have incorporated nanomaterials. Other techniques used to enhance the performance of nanobiosensors include multiplex testing, point-of-care testing (POCT), and smart sensing.

**Methodology:**

Thus, in this review, we present a comprehensive overview of the state-of-the-art nanobiosensors for detecting infectious diseases. The review covers key topics that are centred around the application of nanotechnology in biosensing, multiplex testing, POCT and smart nano-enhanced biosensors.

**Findings:**

The findings of this review highlighted the advantages of biosensors over conventional approaches, with a limit of detection ranging from nanomolar to attomolar concentrations and a time response ranging from 1 to 3 hours.

**Conclusion:**

Despite the prospect of nanobiosensors, several limitations exist, including complexity, extensive processing time, and others. Moreover, the integration of smart technologies in nanobiosensors can offer several benefits, including high accuracy and faster detection and prediction.

## 1. Introduction

Since the beginning of writing, ancient civilizations have recorded many infectious diseases which have impacted people's lives [1]. Several diseases have been recorded throughout history, with some causing global havoc in terms of mortality and fatality [2-3]. The progress made in medical care has led to the classification of diseases into several categories, which include pathogenic diseases, hereditary diseases, deficiency diseases, and psychological diseases [4-5]. Diseases can also be classified into communicable and non-communicable diseases, as well as zoonotic diseases, waterborne diseases, and foodborne diseases [6-7]. Among these types of diseases, infectious diseases caused by pathogens such as viruses, bacteria, fungi and parasites pose a serious problem to global health. Several infectious diseases can be transmitted from one person to another. Over the years, infectious diseases have caused several epidemics and pandemics and have resulted in millions of deaths globally [8-9].

Identifying pathogens responsible for causing an infection is of paramount importance to medical care. Several techniques have been developed over the years, ranging from culturing and microscopy to spectroscopy, serological assays such as enzyme-linked immunosorbent assay (ELISA), lateral flow tests, PCR, and biosensors [10-12]. Despite wide arrays of these approaches, they have several drawbacks which include low sensitivity, low specificity or selectivity, low throughput, false positive results, lack of point-of-care testing (POCT), expensive, longer processing time, laborious sample collection and processing procedures, the need for train expert, contamination and safety concerns, the use of toxic chemicals, requirement of large-scale devices, longer wait time for test results etc. Therefore, there is a need for an effective approach that can address these limitations [10].

The development of the first glucose biosensors has transformed the landscape of biomedical sensors and laid the groundwork for the advancement of biosensors in clinical diagnosis of infectious diseases [13]. Biosensors for infectious disease detection have been developed to identify or recognize pathogen-related biomolecules or components using highly sensitive and specific recognition elements such as NA (DNA, RNA, aptamers), antigens and antibodies, enzymes, whole cells or cell organelles. Apart from biorecognition elements, biosensors also require transduction and signal amplification systems, such as optical, electrical, electrochemical, mechanical, *etc*. [14]. Biosensors offer several advantages, which include high sensitivity, miniaturization, ease of operation and low cost [15-16].

The integration of nanotechnology for the development of nanobiosensors or nano-biomedical sensors has emerged as a viable approach to address the limitations of conventional approaches. Nano-modified biosensors have been shown to improve conductivity, diagnostic accuracy, sensitivity, fast response, *etc*. [17-19]. Several nanomaterials possess desirable characteristics, including small size, excellent electrical conductivity, high dispersity, large surface area, optical properties, physicochemical properties, and ease of functionalization, which make them ideal for biosensing technologies. Several nanoscale structures have been incorporated in biomedical sensors for detection of infectious diseases, some of which includes Carbon-based NMs such as graphene and its derivatives such as graphene oxide (GO), reduced GO, nitrogen-doped graphene, carbon black (super P), metallic nanoparticles (NPs) such as copper, gold, palladium, aluminium, cobalt, iron, metallic oxides such as zinc, manganese, zirconium and titanium oxides, carbon-based nano-structurers, quantum dots, magnetic NPs, polymeric nanomaterials, nanofibers, fluorescent nano clusters *etc*. [16,20]. Moreover, the integration of nanotechnology in biosensing applications offers opportunities for the fabrication of cheaper, faster and more sensitive biosensors [18-19].

Another challenge facing the diagnosis of infectious diseases includes the lack of POCT and multiplex testing [21-22]. The emergence of infectious diseases in rural areas and underdeveloped countries requires the collection of samples from the site of infection and subsequent transportation to countries with standard medical facilities, which increases cost, processing time and increases the likelihood of contamination and false positive results. Therefore, there is a high demand for POC-based biosensors that can enable onsite detection [21,23,24]. Consequently, as the number of infectious diseases surges coupled with the advancement of the healthcare system, medical laboratories require more effective and cost-effective procedures to manage their increasing workload [15,25]. To address this issue, multiplex testing has emerged as a critical approach and is currently being adopted in clinical practices [26-27]. To enhance the performance of nanobiosensors, scientists have started incorporating cutting-edge technologies such as wireless technology, internet of things (IoT) or internet of medical things (IoMT), artificial intelligence (AI), cloud computing, big biomedical data (BBD), 3-D printing, *etc*. The prospect of smart nanobiosensors is gaining interest due to their potential for real-time detection, disease monitoring, surveillance, and epidemic control, among other applications [14,16,28]. Thus, the continuous need for fast and effective triage of patients, identification of individuals infected with a specific disease, vulnerable individuals, and those at risk, as well as tracking of infection progression, warrants the use of smart technology [14,29].

### 1. 1. Comparison with related review

The review reported by Dhahi *et al.* [12] shares a close similarity with our review, focusing on nanobiosensors for infectious diseases. The review covers several key areas, including background on infectious diseases, outbreaks, modes of transmission, impacts, challenges, and a gateway to nanobiosensors, as well as future directions and conclusions. However, the review does not include the detailed prospects of smart technologies, multiplex testing and POCT.

Another review discussing the application of nano-modified sensors for detecting infectious diseases is presented by Rios *et al.* [19]. The review outlines key issues, including nanosensors, antibody-based, NA-based, and peptide-based applications, as well as agricultural and industrial applications, future perspectives, and conclusions. The review differs from our review based on the scope (animal infectious diseases vs human infectious diseases). Moreover, the review does not include the potential of smart nanobiosensors for multiplex and POCT. Similarly, the review presented by Singh *et al.* [18] focuses solely on nanobiosensors and their applications in infectious disease management.

The review presented by Irkham *et al.* [11] shared a close similarity with this review in terms of nanotechnology and clinical diagnostics. Despite the shared scope, the review is limited to graphene-based electrochemical nanobiosensors. Moreover, the study does not include multiplex testing. The review reported by Kerry *et al.* [7] highlighted the application of nanotechnology for the detection of non-communicable and communicable diseases. The study covers several topics, including detailed information on biosensors, application of nanotechnology in biosensing, application of nano-integrated biosensors for detection of communicable diseases (viruses, fungi, bacteria, parasites) and non-communicable diseases (Parkinson's, Alzheimer's, neonatal disorders, bilirubin), commercialization of nanobiosensors and future prospects. Smart, multiplex, and POCT do not fall within the scope of the study. The summary of the comparison with the related review is presented in Table 1.

**Table 1.** Comparison with related review

Biosensors	Nanotechnology	POCT	Multiplex testing	Smart technology	Open research	Ref.
✓	✓	X	X	X	✓	[12]
✓	✓	X	X	X	✓	[19]
✓	✓	X	X	X	✓	[18]
✓	✓	✓	X	X	✓	[11]
✓	✓	X	X	X	X	[7]
✓	✓	✓	✓	✓	✓	This study

## 2. Methodology and objectives

Rapid, ultra-sensitive, ultrafast, simple, free amplification, free sample pre-treatment, POC and multiplex testing have long been key objectives for medical practitioners. Therefore, the main focus of this review revolves around the application of nanotechnology in biosensing techniques for POCT, multiplex and smart detection of pathogenic diseases. The main idea behind the formulation of the research questions stemmed from the scarcity of comprehensive reviews on the subjects and the pressing need, underlined by the emergence of pathogenic diseases and the limitations of conventional approaches. The research questions include:

- How do nano-modified biosensors enhance the sensitivity and specificity of pathogen detection compared to traditional diagnostic methods?
- How can nano-modified biosensors be optimized for POCT to enable rapid, accurate, and user-friendly diagnosis of pathogenic diseases in resource-limited settings?
- What are the current or key challenges in developing smart, multiplexed, and point-of-care nano-modified biosensors for the detection of pathogenic diseases?

### 2. 1. Literature search strategy and inclusion criteria

There is a plethora of review articles that cover the application of nanotechnology in biosensing technologies. However, there is no recent review that hyphenates biosensors, nanotechnology, POCT, multiplex testing and smart technology. The initial search for articles used in this review was based on Google Scholar search using keywords such as biosensors, nanotechnology, nano-biosensors, nano-modified sensors, detection of pathogenic disease (specific to a pathogen) using nano-enhanced biosensors, electrochemical-based biosensors for detection of pathogenic disease, POC-based biosensors, multiplex detection, smart biosensors, *etc*. For basic information regarding subjects such as nanotechnology, biosensors, and infectious diseases, we relied on articles retrieved from Google Scholar and the Web of Science. While for research articles that reported the development of nano-modified biosensors for the detection of specific pathogens, we only relied on articles retrieved from Web of Science (Emerging Source Citation Index, (ESCI), Science Citation Index (SCI) and Science Citation Index Expanded (SCIE)). We focused on articles published within the 10-year span (*i.e.* 2015-2025).

We retrieved 238 articles from both Google Scholar and Web of Science. We selected 96 papers published in various outlets, including Springer, ScienceDirect, and Taylor & Francis. The main selection criteria prioritized articles that undergo peer review, have strong scientific content, and are written in English.

### 2. 2. Scope

This review article aims to provide an overview of the prospects of the integration of nanotechnology and smart technology for the development of biosensors for the detection of infectious diseases. The review is structured using key topics, which include an overview of biosensors, working principles, and types (based on bioreceptors and transduction). The state-of-the-art studies that reported the development of nano-modified biosensors for infectious disease detection are also outlined. Moreover, the concept of smart technology, which includes wireless and IoT systems, AI, and BBD, is also highlighted.

The remaining part of this review is organized as follows. Section 3 reviews existing studies that have reported the use of nanotechnology to enhance biosensing capabilities for the detection of infectious diseases. Moreover, the chapter also covers multiplex testing and the prospect of smart technologies in biosensing. Section 4 highlights the key findings and open research issues, and lastly, Section 5 constitutes the conclusion.

## 3. Applications of nanobiosensors for the detection of pathogenic diseases

### 3. 1. Pathogenic diseases

Pathogenic diseases are characterized by infections caused by various microorganisms, which include bacteria, viruses, fungi and parasites [15]. Pathogenic diseases can be categorized into two classes based on their spread, which include communicable and non-communicable diseases. Communicable diseases are the type that can be transmitted from a host or reservoir to a healthy person. Some of the transmission approaches include direct contact, animal-to-human transmission, water contamination, air contamination, and food contamination. Pathogenic diseases induce a range of symptoms, varying from acute to critical or life-threatening [15,30,31].

#### 3. 1. 1. Viruses

Pathogenic viruses are responsible for causing several diseases and contribute to morbidity and mortality every year. Example of pathogenic viruses include Ebola which caused haemorrhagic fever, hepatitis, human immunodeficiency virus/acquired immunodeficiency syndrome (AIDS), SARS-CoV-1, SARS-CoV-2, Middle East respiratory syndrome (MERS), Dengue which caused dengue haemorrhagic fever (DHF) and dengue shock syndrome (DSS), Zika, Influenza, Yellow fever virus, Rotavirus etc. Viruses are characterized as the smallest disease-causing agents known to humans [10,15,32]. Unlike bacteria, viruses store their genetic information in the form of RNA and require a host cell to replicate through hijacking host replication machinery, resulting in the production of millions of new viral particles [33]. Several viral diseases are associated with severe symptoms, which necessitate proper disease management.

#### 3. 1. 2. Bacteria

The world has been battling against pathogenic bacteria, which continue to pose serious health concerns. Bacterial infections are responsible for millions of deaths worldwide. Currently, there are several pathogenic bacteria which pose serious health issues; some of these bacteria include *Staphylococcus aureus* (*S. aureus*) associated with gastrointestinal tract disease, shock syndrome and septicemia [34], *Mycobacterium tuberculosis, Salmonella, Escherichia coli, Streptococcus pneumonia, Vibrio cholerae*, *etc*. [35]. Unlike viruses, store their genetic information in the form of DNA, which is typically a single circular chromosome [36]. Bacteria can be categorized based on their shapes, such as spherical (cocci), rod-shaped (bacilli) and spiral shape (spirilla) [37-38]. Moreover, bacteria can be categorised into Gram-positive and Gram-negative based on the staining technique. Gram-negative bacteria are characterized as bacteria that do not retain violet stain and typically appear red or pink under the microscope. Gram-positive bacteria are characterized as those that retain violet stain and appear purple under the microscope [39].

### 3. 2. Application of nano-modified biosensors for the detection of viruses

Over the last 2 decades, several studies have reported the development of nano-modified based biosensors for the detection and quantification of pathogenic diseases which include the recent SARS-COV2, Dengue, Zika, papillomavirus (HPV), HIV, Ebola, Chikungunya, Influenza, SARS-CoV-2, HIV, Hepatitis B and C, Zika, Ebola, as well as the need for adaptable and fast-deploying nanobiosensors and potential for point-of-care (POC) diagnostics [7,11,12,18,19].

The development of rapid, sensitive, POC and early detection of Ebola virus disease (EVD) using nano-modified immunosensor is presented by Chen *et al.* [23]. The biosensor is designed based on a reduced graphene oxide-based field-effect transistor (FET) method. The detection criteria rely on the rGO as a conducting channel electrode, which is thermally annealed on the electrode through chemical absorption. An Al_2_O_3_ layer and gold nanoparticles are also incorporated on top of the FET. The channel is subsequently immobilized with anti-Ebola probes that can specifically capture the target antigen. Evaluation of the biosensor has shown that it can successfully detect the target (Zaire strain) down to a LOD of 1 ng mL^1^. To evaluate selectivity and sensitivity, two viral samples, which include Marburg and Sudan viruses, are used as control samples.

Kaushik *et al.* [24] developed an electrochemical-based immunosensor for the detection of ZIKV. The biosensing strategy revolves around the functionalization of interdigitated microelectrodes of gold (IDE-Au) and immobilization of ZIKV-specific envelop protein antibody onto dithiobis(succinimidyl propionate). The resulting interaction between the immobilized antibody and target ZIKV was measured based on electrochemical impedance spectroscopy (EIS). Evaluation of the developed electrochemical-based immunesensor resulted in an LOD of 10 pM and a detection range from 10 pM to 1 nM. The developed biosensor has the potential for POCT through the integration of the biosensing chip with a miniaturized potentiostat (MP)-interfaced with a smartphone.

Zang *et al.* [40] reported the development of a nano-modified immunosensor for the rapid and ultra-sensitive detection of Ebola virus (EBOV) antigens. The biosensor is a 3D plasmonic nanoantenna assay sensor consisting of a silicon dioxide nanopillar array, Au nanodisk, Au nanodots, and Au backplane, which exhibits substantial fluorescence intensity enhancement. The nanoantenna array immunosensor comprises a thin layer of plasmonic nanoantenna array with its surface connected to a molecular linker layer. EBOV capture agents are used to detect two Ebola antigens, which include EBOV sGP and EBOVgp-Fc, via a sandwich assay protocol. The nanoantenna-based immunosensor can successfully detect EBOV soluble glycoprotein (sGP) in human plasma down to an LOD of 220 fg mL^-1^. Side-by-side comparison using ELISA was also conducted to verify the performance of the developed biosensor.

Chowdhury *et al.* [41] developed an electrochemical-based nanobiosensor for the detection of the Hepatitis E virus. The electrode of the biosensor was fabricated using graphene quantum dots (GQDs) and gold-embedded polyaniline nanowires coupled with the immobilization of anti-HEV antibody. To increase sensitivity, the study introduced an external electrical pulse during virus accumulation. The nano-electrochemical-based biosensor was deployed to detect specific HEV genotypes (G1, G3, G7 and ferret HEV). Consequently, the developed framework is compared with real-time quantitative reverse transcription-polymerase chain (RT-qPCR), which resulted in similar sensitivity. Assessment of the developed biosensor has shown that it can detect HEV with a wide linear range of 1 fg mL^-1^ to 100 pg mL^-1^ and a low detection limit of 0.8 fg mL^-1^ under optimal conditions. Moreover, to assess the sensitivity and specificity of the developed nanobiosensor, it is tested using HEV-like particles (HEV-LPs) in buffer and human serum.

The development of an electrochemical-based immunosensor for the multiplex detection of MERS-CoV and human coronavirus (HCoV) is reported by Layqah and Eissa [42]. In order to accomplish rapid and accurate detection of the target protein, the biosensor is fabricated by functionalizing carbon electrodes with AuNPs and subsequent immobilization of recombinant spike protein as a biomarker for MERS-CoV. The multiplexed detection of different MERS-CoV is owed to the utilization of arrays of electrodes. The biosensing approach revolves around the indirect competition between free virus and immobilized MERS-CoV protein for a fixed concentration of antibody incorporated into the sample. Assessment of the developed nano-modified immunosensor has shown that it can be conducted in 20 min with LOD of 0.4 and 1.0 pg mL^-1^ for HCoV and MERS-CoV, respectively and linear response from 0.001-100 ng mL^-1^ and 0.01-10,000 ng mL^-1^ for MERS-CoV and HCoV, respectively.

**Figure 1.**
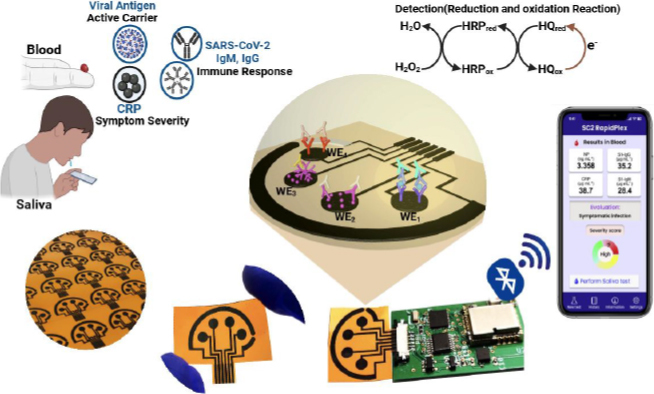
SARS-CoV-2 RapidPlex. A-D. Comprises of WEs: (4 graphene); RE: Ag/AgCl; CE: Graphene. All the electrodes (6: 4 WEs and 1 RE and 1 CE) are patterned on a polyimide (PI) substrate via CO_2_ laser engraving. Detection of target proteins and specific immunoglobulins via sandwich and direct immunosensing approaches onto laser-engraved graphene (LEG) electrodes

As a result of the need for a rapid technique that supports widespread testing to mitigate the spread of SARS-CoV-2, Torrente-Rodríguez *et al.* [43] developed a graphene-based multiplexed telemedicine platform known as SARS-CoV-2 RapidPlex for rapid, ultra-sensitive and low-cost detection of COVID-19 using saliva and serum samples. The platform is designed based on target-specific immunoassays constructed using laser-engraved graphene for the rapid and remote assessment of SARS-CoV-2 biomarkers, including anti-spike protein IgG and IgM, nucleocapsid protein, and C-reactive protein (CRP). Laser-engraved graphene (LEG) undergoes functionalization and modification steps for the covalent attachment of receptors. The multiplex detection of target biomarkers provides information that includes viral infection, immune response, and disease severity. The assessment of the immunosensor using serum and saliva resulted in concentration range from 0.1-0.8 μg mL^-1^ and 0.5-2.0 ng mL^-1^ for nucleocapsid protein, 20 to 40 μg mL^-1^ and 0.2 to 0.5 μg mL^-1^ for S1-IgG, 20 to 50 μg mL^-1^ and 0.6 to 5.0 μg mL^-1^ for S1-IgM and 10 to 20 μg mL^-1^ and 0.1 to 0.5 μg mL^-1^ CRP. The summary of the SARS-CoV-2 RapidPlex platform is presented in Figure 1.

Siew *et al.* [44] reported the construction of a high-performance immunosensor fabricated with graphene/titanium dioxide (G/TiO_2_) for the detection of Dengue virus IgG antibodies. The experiment was set up using a screen-printed carbon electrode (SPCE) modified with a G/TiO_2_ nanocomposite and the Plant-derived dengue envelope domain III (EDIII) protein as an antigenic probe. Evaluation of the immunosensor displays excellent results in terms of specificity (discriminating between dengue and Zika virus) and sensitivity with LOD of 2.81 ng mL^-1^ and a wide linear range from 62.5-2000 ng mL^-1^. The results also indicate consistency with those obtained using ELISA with mouse serum samples.

Lee *et al.* [45] developed an electrochemical-based genobiosensor enhanced with gold NPs for the ultra-sensitive detection of Dengue virus. The biosensor is designed based on the use of methylene blue-functionalized Au NPs coupled with CRISPR/Cpf1 (Cas12). The detection process revolves around the deployment of CRISPR/Cpf1, which recognizes and randomly cleaves target nucleic acid as a result of its nonspecific ssDNA trans-cleavage activity. The developed nano-modified genobiosensor can detect target dengue fever viral samples down to a LOD of 100 fM. The biosensor has demonstrated the ability to detect samples within 30 minutes, eliminating the need for an RNA amplification step.

The development of a cost-effective, simple, ultrafast and rapid biosensing approach for the detection of EBOV is reported by Chen *et al.* [46]. The study developed an assay featuring nanobody-conjugated NPs for rapid, electronic detection (Nano2RED). The detection mechanism revolves around the use of generated nanobodies conjugated to AuNPs for in-solution colorimetric detection of EBOV secreted glycoprotein (sGP) target. Evaluation of the nano-modified optical immunosensor has shown that it can detect with a LOD of 350 pM and a dynamic range from 100 pM to 1 μM. The developed biosensor is also compared with ELISA to establish consistency.

Yadav *et al.* [47] proposed the development of an electrochemical-based immunosensor fabricated using a nanocomposite made of molybdenum disulfide nanosheets decorated with polydopamine (MoS2-PDA) for the detection of SARS-CoV-2 nucleocapsid protein (N protein). Detection of the target revolves around the functionalization of the nanocomposite with anti-SARS-CoV-2 nucleocapsid IgG antibody (Ab), followed by quantification via EIS. The assessment of the fabricated nanocomposite-enhanced immunosensor resulted in a LOD of and 2.80 ag mL^-1^ and a limit of quantification (LOQ) of 8.48 ag mL^-1^ and a linear range from 10 ag mL^-1^ and 100 ng mL^-1^. Moreover, the biosensor was also compared with the results obtained using a conventional RT-PCR test based on the detection of N protein in nasopharyngeal swab specimens, indicating consistency.

Sangili *et al.* [48] developed a label-free electrochemical-based immunosensor enhanced with a nanocomposite for the rapid detection of DENV protein. The biosensing strategy revolves around the in-situ reduction and functionalization of AuNPs decorated with heteroatom-doped reduced graphene oxide nanocomposites coupled with the immobilization of an antibody. The result of the fabricated immunosensor exhibited LOD of 1.6 pg mL^-1^ and a wide range from 0.01 to 100 ng mL^-1^. The platform was also tested for the detection of viral E-protein contents in serum samples and subsequently compared with the results recorded using the ELISA approach.

Del Caño *et al.* [49] reported the development of an amplification-free electrochemical-based genosensor modified using Gold nanotriangles (AuNTs) for the detection of SARS-CoV-2. The biosensing strategy revolves around the functionalization of AuNTs with dithiolated oligonucleotides and the subsequent hybridization with either single-stranded DNA or RNA sequences of SARS-CoV-2 of different lengths, resulting in quantification. The developed electrochemical genosensor can also detect point mutations in the viral genome and discriminate between variants, including Alpha, Beta, Gamma, Delta, and Omicron. The evaluation of the platform resulted in LOD of 22.2fM and LOQ of 75.7 fM. The result achieved is compared with RT-qPCR to ensure consistency. In addition, the genobiosensor is also employed to detect the target in nasopharyngeal swab samples from COVID-19 and has been shown to accurately discriminate between infected and non-infected cases.

Braz *et al.* [50] reported the development of an electrochemical immunosensor fabricated using AuNPs for the detection of SARS-CoV. The nano-modified immunosensor is designed based on solid-binding for the detection of SARS-CoV-2 anti-S antibodies. The mechanism behind the detection procedure revolves around the use of a peptide as a recognition site with two regions. The first region is based on the viral receptor binding domain, which is capable of recognizing antibodies of the spike protein, while the second region is designed for interacting with AuNPs. AuNP-binding peptide dispersion is utilized to modify the screen-printed carbon electrode (SPCE). To evaluate the detection capability of the developed nanobiosensor, differential pulse voltammetry was used. The result indicated that the biosensor could detect the target with a LOD of 30 ng mL^-1^ and a linear working range from 75 ng mL^-1^ to 15 μg mL^-1^.

The development of a digital CRISPR-powered nano-modified genosensor for the detection of DENV is reported by Freko *et* al. [51]. The developed genosensor combined the collateral cleavage activity of the Cas12a enzyme with a digital sensing strategy, based on the oxidation events of DNA-coated AgNPs on a microelectrode array. Evaluation of the nano-enhanced biosensor has demonstrated that it can successfully detect targets at concentrations as low as 25 pM. The biosensor has demonstrated the ability to detect the target within 50 minutes. The application of nano-modified biosensors for the detection of viruses is summarized in Table 2.

**Table 2.** Application of nano-modified biosensors for the detection of viruses

Type of Biosensors	Nanomaterial	Target	LOD	References
Immunosensor	rGO and AuNPs	EBOV	1 ng mL^-1^	[23]
Immunosensor	IDE-Au	ZIKV	10 pM	[24]
Immunosensor	Nanoantenna array	EBOV	220 fg mL^-1^	[40]
Immunosensor	GQDs and AuNP-PAni	Hepatitis E virus	0.8 fg mL^-1^	[41]
Immunosensor	AuNP	MERS-CoV and HCoV	0.4 and 1.0 pg mL^-1^	[42]
Immunosensor	LEG	SARS-CoV-2	0.1–50 μg mL^-1^	[43]
Immunosensor	G/TiO^2^	DENV	2.81 ng mL^-1^	[44]
Genosensor	AuNPs	DENV	100 fM	[45]
Immunosensor	AuNPs	EBOV	350 pM	[46]
Immunosensor	MoS_2_-PDA	SARS-CoV-2	2.80 ag mL^-1^	[47]
Immunosensor	AuNPs	DENV	1.6 pg mL^-1^	[48]
Genosensor	AuNTs	SARS-CoV-2	22.2 fM	[49]
Immunosensor	AuNPs	SARS-CoV-2	30 ng mL^-1^	[50]
Genosensor	AgNPs	DENV	25 pM	[51]

### 3. 3. Application of nano-modified biosensors for the detection of bacteria

Kaur *et al.* [52] proposed the construction of Bridged Rebar Graphene (BRG) nanostructured aptasensor for the rapid detection of pathogenic *E. coli*. The proposed biosensor was designed based on the functionalization of BRG onto the fabricated nanostructured aptasensor. The biosensing strategy revolves around bacteria-DNA interactions captured on the BRG nanostructured electrode by using specific anti-E. coli DNA aptamer. Evaluation of the performance of the developed nano-modified aptasensor resulted in LOD of approximately 10 CFU mL^-1^ with a dynamic response range from 10^1^ to 10^6^ CFU mL^-1^ in 3 samples, which include milk, water and juice.

Zhang *et al.* [53] reported the development of an enzyme-free electrochemical-based biosensor for the detection of *Pseudomonas aeruginosa*. The biosensor was fabricated using ZrMOF, which was subsequently connected with Cu^2+^ to form Cu-ZrMOF, possessing high catalytic activity. Moreover, a nanocomposite composed of Cu-ZrMOF@Aptamer@DNA served as a signal probe to catalyse the decomposition of H_2_O_2_. Furthermore, to increase the electron transfer, super P was incorporated. In order to test the biosensor using a clinical sample, a spiked urine sample is used to quantify the bacteria. The developed nanocomposite-based biosensor exhibited a wide linearity range of 10 to 10^6^ CFU mL^-1^, with a LOD of 2 CFU mL^-1^. The biosensor has been shown to be more sensitive compared with other conventional methods.

**Figure 2.**
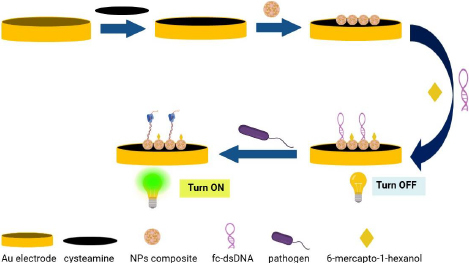
Electrochemiluminescence biosensing platform for *Listeria monocytogenes*

The development of an electrochemiluminescence aptasensor enhanced using ruthenium(II) tris-(bipyridine) Ru(bpy)_3_^2+^-AuNPs for the detection of *Listeria monocytogenes* is proposed by Cui et al. [54]. The aptasensor is designed based on two parts, which include an electrochemiluminescence (ECL) substrate and an ECL intensity switch. The first part revolves around the modification of the Au electrode using AuNPs and Ru(bpy)_3_^2+^, while the second part is characterized by the use of ferrocene-labelled molecular beacon aptamer (Fc-MBA), which acted as an electrochemiluminescence intensity switch, as shown in Figure 2. Analysis of the performance of the biosensor resulted in a LOD of 5 CFU mL^-1^ and a linear range from 7 to 7×10^6^ CFU mL^-1^. The electrochemiluminescence aptasensor is also tested to analyse its specificity using other bacteria, including *Bacillus subtilis, Shigella flexneri, Salmonella*, *E. coli* and *S. aureus*. Moreover, the aptasensor is also used to determine the bacteria in milk samples.

The development of an in situ electrochemiluminescence-based aptasensor for the detection of *S. aureus* is reported by Liu *et al.* [34]. The biosensing technique relies on the hybridization between multi-DNA strands with captured DNA assembled on an electrode modified with Ru(bpy)_3_^2+^-conjugated silica NPs (RuSi NPs). Subsequently, glucose oxidase (GOD) was introduced through the functional conjugation of the enzyme with the multi-DNA, where the catalysis of GOD on glucose was accompanied by the presence of hydrogen peroxide. The ECL quenching of H_2_O_2_ in the Ru(bpy)_3_^2+^ system led to the quantification of the bacteria. The analysis of the aptasensor resulted in a LOD of 3.0 CFU mL^-1^ and a linear range from 10 to 10^7^ CFU/mL.

Sohouli *et al.* [55] proposed the development of a nano-enhanced electrochemical-based aptasensor for the detection of *S. aureus*. The aptasensor was designed based on the modification of screen-printed carbon electrodes (SPCE) with nitrogen-doped carbon nano-onions, AuNPs, followed by self-assembly of the aptamer on the SPCE surface through covalent modification of the thiol group with gold NPs. Moreover, in order to improve the analytical parameters of the aptasensor, a carbon layer was immobilized on the electrode surface. Evaluation of the analytical performance of the aptasensor resulted in a LOD of 3 CFU mL^-1^ and a linear range from 10 to 10^8^ CFU mL^-1^.

Abedi *et al.* [56] reported the development of a nano-modified impedimetric-based aptasensor for the ultra-sensitive detection of *Acinetobacter baumannii* bacteria. The miniaturized electrochemical biosensor leverages a label-free detection approach by exploiting EIS. The biosensing strategy revolves around the immobilization of an aptamer on the surface of CSPE modified with nanocomposite Fe_3_O_4_@SiO_2_@Glyoxal (Gly), which resulted in signal amplification. The evaluation of the label-free nano-modified aptasensor's performance revealed an LOD of 150 CFU/mL and a detection range of 10^3^ to 10^8^ CFU mL^-1^. The developed biosensor also yields satisfactory results when used for detecting bacteria in blood serum samples.

To rapidly and accurately detect *E. coli*, Zhong *et al.* [57] reported the construction of a nano-enhanced electrochemiluminescence aptasensor. The advantage of the developed biosensor revolves around the use of a double aptamer recognition system, which resulted in increased accuracy. The biosensing approach revolves around the use of nano-cadmium sulphide (CdS) modified aptamer for primary labelling and subsequent immobilization of the second aptamer with a graphene/chitosan composite electrode for re-capture. Evaluation of the performance of the nano-enhanced electrochemiluminescence aptasensor resulted in a LOD of 10 CFU mL^-1^ and a linear range from 10^2^ to 10^7^ CFU mL^-1^.

Yadav *et al.* [58] reported the development of a nano-enhanced electrochemical-based aptasensor for the detection of *H. pylori*. The platform is designed based on 3-(aminopropyl) triethoxysilane (APTES), an organofunctional silane used to functionalize graphitic carbon nitride (g-C_3_N_4_), which is subsequently applied to screen-printed electrodes (SPE). A Cag A-specific aptamer is covalently immobilized on the APTES@g-C3N4/SPE. The assessment of the developed nano-enhanced electrochemical-based aptasensor demonstrated an LOD of 0.017 ng mL^-1^, LOQ of 0.056 ng mL^-1^ and linear detection range from 0.1 to 160 ng mL^-1^. Moreover, the biosensor also exhibited a high sensitivity of 1.98 μA ng mL^-1^ cm^-2^.

Jaradat *et al.* [59] constructed an electrochemical-based immunosensor for the selective detection of the HopQ biomarker of H. pylori bacteria. The proposed biosensor was constructed by surface-modifying SPCE with multi-walled carbon nanotubes (MWCNT)-COOH and subsequently decorating it with AuNP. The nano-modified electrodes are subsequently immobilized with HopQ capture antibody grafting. To analyse the performance of the sensor, several techniques were employed, including EIS and cyclic voltammetry (CV). The result indicated that the electrochemical-based nano-modified immunosensor achieved an LOD of 2.0 pg mL^-1^, LOQ of 8.6 pg mL^-1^ and linear range from 10 pg mL^-1^ to 100 ng mL^-1^. The platform is also used to detect *H. pylori* in spiked saliva samples and further analysed using square wave voltammetry (SWV).

Wang *et al.* [60] developed a one-step label-free colorimetric-based nano-enhanced immunosensor for the detection of *Vibrio parahaemolyticus*. The overall techniques revolve around utilization of phage-Nbs specific to V. parahaemolyticus, followed by thiolation in which the thiol group on the phage surface induce aggregation of AuNPs; hence, the interaction with *V. parahaemolyticus* prevents the aggregation, resulting in changes of solution colour and the optical intensity spectrum. Assessment of the biosensor based on LOD and quantitative detection limit resulted in 10^4^ cfu/mL and 10^3^ cfu/mL, respectively.

The development of electrochemical-based immunosensors modified using GO or graphene quantum dots (GQD) for rapid detection of leishmaniasis is proposed by Braz *et al.* [61]. To detect bacteria in human and canine serum samples, the study employed different peptides that share the same recognition site but differ in their solid-binding domains. The biosensor is also evaluated against *Mycobacterium tuberculosis* and *Mycobacterium leprae*, and the immunosensor displays selective behaviour against both bacteria.

Hu *et al.* [62] reported the development of an electrochemiluminescence-based approach capable of both DNA analysis and genotype classification of H. pylori. The biosensor is fabricated using AuNP and CdS as a signal switch. The strategy revolves around the use of a Y-shaped structure to capture the target DNA sequence with all the genotypes, resulting in an electrochemiluminescence signal. Subsequently, Cas9 is used to cleave the Y-shaped structure with the concomitant signal decrease. Thus, the combination of these approaches characterized by different selectivity can be used to quantify the total amount of bacterial DNA and the ratio of single-nucleotide variants (SNV). Moreover, the developed strategy is also used to conduct a large-scale screening assay on a chip. Analysis of the biosensing strategy has resulted in LOD of 8 pM and linear range from 0.01-500 nM. The selectivity of the developed biosensor toward *H. pylori* was compared with that of other bacteria, including *E. coli, Shigella, Salmonella* and *S. aureus*. The application of nano-modified biosensors for the detection of bacteria is summarized in Table 3.

**Table 3.** Application of nano-modified biosensors for the detection of bacteria

Type of biosensors	Nanomaterial	Target	LOD	Ref.
Aptasensor	Bridged Rebar Graphene (BRG)	*E. coli*	101 CFU/mL	[52]
Aptasensor	Cu-ZrMOF	*Pseudomonas aeruginosa*	2 CFU mL^-1^	[53]
Aptasensor	Ru(bpy)_3_^2+^-AuNPs	*Listeria monocytogenes*	5 CFU/mL	[54]
Aptasensor	RuSi NPs	*Staphylococcus aureus*	3.0 CFU mL^-1^	[34]
Aptasensor	nitrogen-doped carbon nano-onions, AuNPs	*Staphylococcus aureus*	3 CFU/mL	[55]
Aptasensor	Fe_3_O_4_@SiO_2_@Glyoxal (Gly)	*Acinetobacter baumannii*	150 CFU/mL	[56]
Aptasensor	nano-cadmium sulfide	*E. coli*	10 CFU mL^-1^	[57]
Aptasensor	g-C_3_N_4_	*H. pylori*	0.017 ng mL^-1^	[58]
Immunosensor	MWCNT-COOH and AuNP	*H. pylori*	2.0 pg/mL	[59]
Immunosensor	AuNPs	*Vibrio parahaemolyticus*	10^4^ CFU/mL	[60]
Immunosensor	GO or GQD	*Leishmania*	N/A	[61]
Genosensor	AuNPs-CdS	*H. pylori*	8pM	[62]

### 3. 4. Application of nano-modified biosensors for the Point-of-care testing of pathogenic diseases

Early and timely detection of infectious diseases is crucial for effective treatment and medical cost reduction. Therefore, it is very crucial to have an ultra-sensitive, rapid, robust, selective, and specific detection approach. The healthcare sector is highly in need of miniaturized, portable or deployable detection techniques that can replace the heavy-mounted and time-consuming laboratory-based assays. One of the technologies that fulfils these criteria is POC technology. POC testing is driven by biosensors, which offer selective and sensitive analysis of biochemical recognition [21,63]. Biosensors offer several advantages, including portability, specificity, affordability, and rapid detection, compared to conventional techniques. There are several transduction mechanisms used in biosensing technology, dominated by electrochemical and optical approaches. Electrochemical-based biosensors (conductometric, voltammetric, potentiometric, amperometric, and impedimetric) offer several benefits, including speed, accuracy, sensitivity, practicability, and affordability [17,63].

Biosensors are regarded as ideal devices for POCT, owing to their portability. A limited number of studies have reported the development of POC-based biosensors. For example, the prospect of a nano-modified immunosensor for POCT of Ebola virus, evaluated using viral Glycoprotein diluted in PBS buffer, human serum, and plasma, is presented by Chen *et al.* [23]. To enable POC detection, Chen *et al.* [21] combined the assay with a portable semiconductor device, a digital display, and a minimal training requirement for end-users. This is achieved by using a cost-efficient portable UV-visible spectrometer system. Moreover, the study also developed a homemade LED-photodiode-based electronic readout system in order to enable accurate and sensitive detection. The development of a POC-based electrochemical immunosensor by Kaushik *et al.* [24] relies on the introduction of miniaturized potentiostat (MP)-interfaced with a smartphone for rapid and online detection of ZIKV. Thus, the successful integration of nano or microelectronics in the development of miniaturized potentiostat resulted in a smart biosensor capable of POC application.

### 3. 5. Application of nano-modified biosensors for the multiplex testing of pathogenic diseases

One of the key aspects of the healthcare sector that is integral for disease management is the ability to track and mitigate the spread of pathogenic diseases. This issue can be addressed through the continuous development of novel, rapid, ultrafast, ultra-sensitive, POC and multiplex testing methods that can address the diverse health needs of patients [26-27]. The development of multiplex testing is transforming medical diagnostics as it enables the detection of diseases in patients presenting with multiple symptoms. It also helps medical laboratories to conserve resources compared to single-use testing. Multiplexed testing can be characterized by the use of biosensors for the detection of several variants or strains of pathogens. Multiplex testing enables the simultaneous analysis of samples for multiple pathogens. Moreover, Multiplex testing offers several benefits to the healthcare sector, as it enables hospitals to triage patients better and allocate resources.

**Figure 3.**
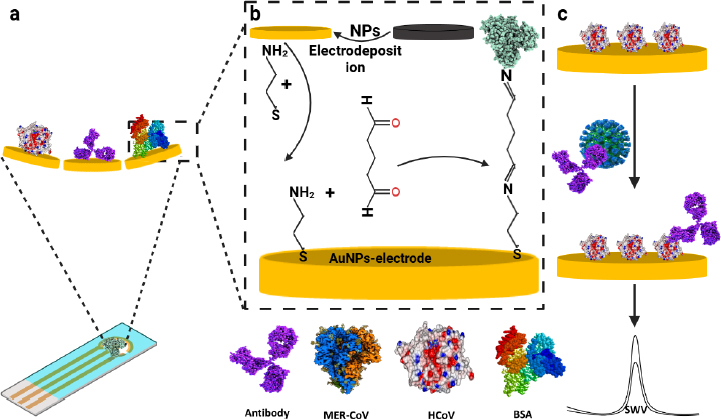
Preparation and detection process of immunosensor array chip for the detection of MERS-CoV. (a) Fabrication steps, (b) Detection process, (c) Response based on monitoring the change in the peak current at different concentrations of the antigen

Despite a plethora of studies on the application of biosensing technologies for the detection of infectious diseases, few studies have reported multiplex testing. For example, the study reported by Layqah and Eissa [42] demonstrated the use of an electrochemical-based immunosensor to detect MERS-CoV and HCoV, utilising an array of carbon electrodes modified with AuNPs, as illustrated in Figure 3. Torrente-Rodríguez *et* al. [43] have demonstrated how SARS-CoV-2 RapidPlex can be used to detect several SARS-CoV-2 biomarkers by using 4 different WEs.

### 3. 6. Application of smart technology in biosensing

The integration of smart technology in the development of nano-modified biosensors has the potential to significantly advance the detection of pathogenic diseases by offering wireless data transfer, cloud-based data storage, and advanced data analysis. As nanotechnology contributes to the development of biosensors with higher sensitivity, specificity and fast response, smart technology enables real-time detection, remote screening, and data analysis [16,64]. The concept of smart biosensors is driven by cutting-edge technologies, including wearable sensors, wireless networks, and smartphone technology. Wearable biosensors are primarily used to measure physiological variables, including temperature, heart rate, blood pressure, oxygen saturation, and blood glucose levels. However, they are very limited for the detection of infectious diseases. The need for remote healthcare monitoring and real-time detection is gaining weight due to the increased likelihood of pandemics and epidemics [65].

The development of smart nano-modified biosensors fabricated into portable or handheld devices can be used for real-time and remote detection, especially in remote or resource-limited settings. The integration of AI and IoT into nano-modified biosensors has the potential to enable the real-time tracking of pathogenic disease outbreaks. AI-driven algorithms such as ML and DL models can be used to analyse data obtained from nano-modified biosensors in order to identify underlying patterns in data and result in classification and prediction of diseases or outbreaks with high accuracy [64,66]. Moreover, cloud-based platforms can also be used to store a large amount of data for analysis using AI tools. Few studies have reported the implementation or integration of smart technologies for improving diagnostic capabilities. Among these studies, Torrente-Rodríguez *et al.* [43] have shown the prospect of electrochemical-based immunosensors integrated with telemedicine for the rapid and multiplexed detection of SARS-CoV-2.

## 4. Key findings, challenges and limitations of nanobiosensors and future directions and emerging trends

### 4. 1. Key findings

#### 4. 1. 1. Nanobiosensors *vs.* conventional biosensors

Despite the advantages of conventional biosensors over laboratory bench assays in terms of portability, simplicity and ease of use, they are not without some drawbacks. Nano-modified biosensors offer several advantages over conventional biosensors. Some of these advantages include enhanced sensitivity, achieved by amplifying signals and facilitating the detection of pathogens at extremely low concentrations. Nanotechnology also contributes to improved specificity via the use of functionalized NPs designed to bind specifically to pathogens [16,17,18,67].

#### 4. 1 .2. Electrochemical *vs.* optical

Optical and electrochemical-based approaches are the common techniques used in biosensing technology. Each type has its own strengths and limitations. Optical-based biosensors offers several advantages over electrochemical-based biosensors, especially in terms of sensitivity due to their light-based detection (fluorescence and surface plasmon resonance) which can detect small changes in wavelength, intensity or refractive index, unlike electrochemical approach which relies on electrical signals generated by redox reaction characterized by lower sensitivity especially for detection of target in complex biological samples [68-71]. Optical-based biosensors also offer label-free detection where the target can be detected without the need for labelling, which also contributes to minimizing preparation time and thereby simplifying the assay [72,73]. Notwithstanding, optical-based biosensors are also suitable for multiplexing as they can be used to detect multiple targets by using different wavelengths compared with the electrochemical approach, which offers several limitations for multiplexing due to the need for complex electrode design, overlapping of signals or sequential measurements [73-75]. Other advantages of optical-based biosensors over electrochemical types include minimal sample preparation, non-destructive and non-invasive nature, reduced susceptibility to interference, and visual readouts [70,71,73].

Unlike the electrochemical transduction approach, the optical approach is limited due to its low precision for target quantification, which is crucial for detecting the severity of infection [10]. ECL technique has emerged as a powerful analytical approach due to its optical setup, high versatility, excellent controllability, wide dynamic detection range, low background noise, *etc*. During the ECL process, electrochemically generated species on the electrode surface occur through high-energy electron transfer, forming excited states and subsequently emitting light [15,34]. Electrochemical-based biosensors offer several advantages, which include ease of miniaturisation, rapid response, high sensitivity and extremely low LODs, low-cost, mostly not requiring complex sample pre-treatment and potential for POCT [63,76]. On the other hand, the main disadvantage of electrochemical-based biosensing approach revolves around instrumental complexity compared to colorimetric techniques [10]. Comparison between optical and electrochemical biosensors is summarized in Table 4.

**Table 4.** Comparison between optical and electrochemical biosensors

Features	Optical-based biosensors	Electrochemical-based biosensors
Sensitivity	Very high	Moderate to high
Specificity	High	High
Portability	High	High
Real-time monitoring	Yes	Limited
Multiplexing	Yes	Limited
Sample preparation	Minimal	May require sample pre-treatment
Interference resistant	High	Moderate

#### 4. 1. 3. Immunosensors *vs.* genosensors

Immunosensors and genosensors are the two most common biosensors used for the detection of infectious diseases. Immunosensors based on antigen-antibody interaction enable more rapid POC detection due to their simple sample preparation, lack of amplification processes; thus, immunosensors are regarded as an ideal approach and surveillance of pathogenic diseases outside clinical settings [46]. Immunosensors based on antigen-antibody offer several advantages, including simplicity and rapid detection. Even though they are very attractive, they are hindered by several limitations, which include the requirement of high quantities of purified antibodies and virus titers in samples [49,77,78].

In contrast to immunosensors, genosensors or genobiosensors, or DNA/RNA-based biosensors have emerged as an alternative approach for the detection of infectious diseases. Genosensors are analytical devices used for the rapid and sensitive detection of pathogens, including viruses and bacteria. Genobiosensors utilized immobilized DNA or RNA probes, which serve as biorecognition elements and hybridize with target nucleic acid [79-80]. Genosensors offers several advantages, which include high specificity, simplicity, high sensitivity, rapid detection, direct analysis of complex samples, fast results acquisition and prospects for POCT. Among several classifications of biosensors, which include electrochemical, piezoelectric and optical biosensors, electrochemical-based biosensors are the most predominant types in the literature [15,80].

#### 4. 1. 4. Nanomaterials

A plethora of studies have reported the integration of nanotechnology for enhancing the performance of biosensors. Moreover, extensive studies have emphasized AuNPs and graphene due to their desirable characteristics. For example, gold nanostructures such as AuNPs are extensively used in electrochemical-based biosensors due to their high surface area, excellent conductivity, outstanding catalytic activity and biocompatibility [49,81]. Thus, combining several materials to create nanocomposites has been shown to exhibit superior properties. For example, studies have shown that combining graphene with metal oxide NPs such as titanium dioxide (TiO_2_) improves performance due to synergistic effects [44,82]. Colorimetric-based biosensors have attracted interest from researchers due to their visual readout, simplicity, portability and cost-effectiveness. Among several NPs used for colorimetric assays, AuNPs are the most widely used for biosensing applications (excellent signal indicators) due to their localized surface plasmon resonance (LSPR), biocompatibility, ease of preparation and high extinction coefficient [83-84].

### 4. 2. Challenges, limitations and prospects of nanobiosensors

In recent years, several techniques have been developed and applied for the rapid and sensitive detection of pathogenic organisms. Despite the progress made in recent years, the screening of pathogenic agents still requires rapid, ultrasensitive, and ultrahigh-throughput screening, as well as low-cost biosensors. The concept of ASSURED, as put forward by the WHO, recommend diagnostic devices to be affordable (A), sensitive (S), specific (S), user-friendly (U), rapid and robust (R), equipment-free (E) and deliverable (D) to those in need [85].

Nano-modified biosensors have shown high potential for POCT and multiplexing due to their miniaturization capabilities, high sensitivity and specificity. Despite the progress recorded in the last few years, Nanobiosensors are still limited by several challenges which hinder their widespread adoption for clinical diagnosis and seemingly integration with smart technologies. One of the challenges facing nano-modified biosensors includes low sensitivity and selectivity [11,20]. These can be attributed to interference from complex biological samples such as blood and saliva, which contain numerous biochemical or biomolecules that can affect the performance of the biosensor. Subsequently, achieving ultra-low sensitivity or LOD for real-world samples is still challenging. Secondly, biosensors are still limited for multiplex testing due to cross-reactivity, which can lead to low accuracy and sensitivity. Despite the extensive studies conducted in the field of biosensing technology over the last 7 decades, the majority of POC-based biosensors are limited due to challenges related to automation, quantitative analysis, and interference with other biomolecules [65].

Biosensors continue to face several limitations regarding seamless integration with smart technologies. For example, several biomarkers produce overlapping signals, which can lead to complications in terms of data interpretation [64,86]. Moreover, real-time data analysis and interpretation require advanced computational resources, such as hardware, software, and AI algorithms, which may not be readily available in portable devices or be prohibitively costly. Moreover, Seamless integration with IoT-based devices and smartphones for data transmission is still under development. The use of nanobiosensors integrated with smart technologies such as AI and cloud computing for data transmission and analysis requires a data protection and security system to prevent data theft and ensure confidentiality [14,16,66].

Another challenge facing nano-modified biosensors revolves around fabrication and scalability, which often requires complex manufacturing processes and sophisticated techniques. Complex manufacturing processes have been shown to increase production costs and are challenging to scale for mass production [20,87,88]. To develop ideal nano-modified biosensors integrated with smart technologies for POCT and multiplexing, several limitations must be addressed. Exploring other nanomaterials or their combinations (such as nanocomposites) can improve sensitivity, biocompatibility and durability [89-90]. Moreover, exploring other novel surface functionalization techniques can improve selectivity and minimize interference [91-92]. Another direction involves the development of nano-modified biosensors integrated with microfluidic systems for automated sample handling and analysis. Thus, the development of microfluidic and Lab-on-Chip systems will enable POCT [93-96].

The continuous integration of smart technologies, such as AI-driven systems for real-time data processing, including noise reduction and analysis through pattern recognition, classification, and interpretation, can improve accuracy. Notwithstanding, seamless integration of wireless and IoT systems will enhance connectivity and data sharing with other devices such as smartphones and cloud systems for remote monitoring and surveillance [14,29,64,66,97].

While nano-modified biosensors have shown great promise for POCT and multiplex testing, however, overcoming current challenges and limitations requires continuous research in the field of material science, engineering, data analytics, confidentiality, data protection, and regulatory compliance. Thus, by addressing these challenges, nanobiosensors can become a cornerstone of next-generation diagnostic devices, enabling smarter, faster, accurate and more accessible healthcare solutions.

## 5. Conclusion

Infectious diseases continue to cause global health challenges. Fast and early detection of pathogenic diseases is crucial for preventing outbreaks, appropriate treatment and reducing costs. For clinical diagnosis of pathogenic diseases, the medical field relies on immunoassays, PCR and reverse transcription-polymerase chain reaction (RT-PCR). Despite the high dependency on these approaches, they are hindered by several limitations. Immunoassays are limited by their low sensitivity, whereas PCR-based methods are more sensitive and specific; however, they also have limitations, including false-positive results, the need for trained experts, susceptibility to contamination, and high costs. Biosensors have become the ideal diagnosis approach due to their sensitivity, portability, specificity and potential for POCT compared with conventional approaches.

The continued transformation in nanotechnology has led to their integration in biosensing applications. Several nanomaterials have been employed to fabricate biosensors, including metal NPs, polymeric nanomaterials, and nanocomposites. The integration of nanotechnology in biosensing applications has been shown to amplify the detection signal. Based on the findings from this review, biosensors offer several advantages over conventional approaches, with LOD ranging between nanomolar to attomolar concentration and time response ranging from 1-3 hours. Few studies have reported achieving lower LOD; however, their strategies are quite complex, extensive and require many steps, which increase time, process and cost. The integration of smart technologies in nanobiosensors has been shown to offer several benefits, which include high accuracy, faster detection and prediction. Smart technologies are crucial for early detection, surveillance, prevention of outbreaks, and effective treatment of pathogenic diseases.
